# miR‐18a activates Wnt pathway in ER‐positive breast cancer and is associated with poor prognosis

**DOI:** 10.1002/cam4.3183

**Published:** 2020-06-16

**Authors:** Madhumathy G Nair, Jyothi S Prabhu, Aruna Korlimarla, Savitha Rajarajan, Hari P S, Roma Kaul, Annie Alexander, Rohini Raghavan, Srinath B S, Sridhar T S

**Affiliations:** ^1^ Division of Molecular Medicine St. John's Research Institute Bangalore India; ^2^ Sri Shankara Cancer Hospital and Research Centre Bangalore India

**Keywords:** ER‐positive breast cancer, migration, miR‐18a, Planar cell polarity pathway, Wnt pathway

## Abstract

Despite the established benefits of long‐term endocrine therapy, women with hormone receptor‐positive breast cancer remain at risk for late relapse. The basis of this is multi‐factorial including genetic, epigenetic, and host factors. In this study we have explored the epigenetic regulation of estrogen receptor (ER)‐dependent molecular and cellular phenotype by hsa‐miR‐18a‐5p using well‐established human ER‐positive (ER+) breast cancer cell lines. miR‐18a was overexpressed in MCF7 and ZR‐75‐1 and this led to an increase in the proliferative ability of the cells and concurrently resulted in decreased expression of luminal markers and higher expression of the basal marker, cytokeratin 14. The cells became more migratory with a significant repression of E‐cadherin and activation of the Wnt noncanonical pathway. We observed an activation of the planar cell polarity (PCP) pathway with increased activation of JNK pathway and eventually change in actin dynamics. There was increased F‐actin polymerization in cells with higher expression of miR‐18a. Examination of miR‐18a expression in a set of human ER+ breast cancer specimens showed a negative correlation between miR‐18a and *ESR1* transcripts as well as ER protein. Kaplan‐Meier survival analysis of the cohort stratified by tumor hsa‐miR‐18a‐5p levels produced significant differences in disease‐free survival (log rank *P* < .05). This observation was independently validated in the METABRIC cohort. These data provide support for a role of hsa‐miR‐18a‐5p in altering the proliferative and migratory behavior of ER+ cells and its potential utility as a prognostic marker in clinical ER+ breast cancers.

## Graphical abstract

Through the manuscript, we have tried to explore the epigenetic regulation of estrogen receptor (ER) dependent molecular and cellular phenotype by hsa‐miR‐18a‐5p. High levels of miR‐18a activates the epigenetic pathway of repression of the luminal phenotype through activation of Wnt pathway.

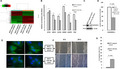

## 1 INTRODUCTION

Breast cancer is the most commonly diagnosed malignancy in women worldwide and the leading cause of cancer death in women.^1^ Hormone receptor‐positive tumors account for around 70% of all invasive breast cancers. ER‐positive breast cancer is known to have a better prognosis compared to the ER‐negative group and ER expression is predictive for responsiveness to endocrine therapies. Although endocrine therapy has improved the survival of ER+ patients considerably, close to a quarter of patients do not respond to endocrine therapies and around 50% of patients acquire resistance to these therapies through multiple mechanisms.^2^, ^3^ Decreased ERα expression and decreased dependency on ER‐based signaling are considered as mediators of endocrine resistance.^4^ The underlying mechanisms behind these observations are not very clear.

MicroRNAs (miRNAs/miRs) are key players involved in the post‐transcriptional regulation of genes and deregulated expression of these microRNAs is implicated in the clinical progression of cancer leading to invasion and metastasis. There exist several reports of microRNAs involved in the process of EMT and metastasis like the miR‐200 family of miRs which are upstream of EMT regulators including ZEB1 and 2.^5^ The miR‐34 family of miRs is known to have a negative feedback regulation to fine‐tune epithelial plasticity during the process of metastasis.^6^ miR‐221 and miR‐222 are also implicated in breast carcinogenesis where they are known to aberrantly activate the process of EMT when upregulated.^7^ ERα functioning is also modulated by microRNAs in breast cancer and the aberrant expression of these miRs is associated with modulating response to endocrine therapy. miR‐22 was found to directly repress ERα and hence found to be downregulated in ER‐positive breast cancer cell lines and clinical specimens.^8^ miR‐221, let‐7, and miR‐206 are examples of other microRNAs that suppress ERα functioning and downstream activity.^7^, ^9^, ^10^ miR‐18a belonging to the miR‐17‐92 cluster is also known to directly repress ERα functioning.^11^ The miR‐17‐92 cluster comprising of miRs that include miR‐17, miR‐18a, miR‐19a, miR‐20a, miR‐19b, and miR‐92a are designated as oncomir‐1 due to their potential role in tumorigenesis in multiple cancer types.^12^ Increased expression of miR‐18a has been associated with nasopharyngeal carcinoma, prostate, and pancreatic cancers.^13^, ^14^, ^15^ In breast cancer it is known to be highly expressed in the triple negative subtype of breast cancer.^16^ It is also known to directly repress ERα translation and thereby block the protective effects of estrogen.^17^

In this study, we have manipulated the epigenetic regulation of ER by microRNA hsa‐miR‐18a‐5p by its overexpression in ER‐positive breast cancer cell lines. We have established a novel molecular mechanism by which high expression of miR‐18a alters the cytoskeletal organization and imparts migratory ability to ER‐positive breast cancer cells. This molecular pathway may form part of the basis of poorer prognosis in ER‐positive tumors with high levels of miR‐18a expression.

## 2 MATERIALS AND METHODS

### 2.1 Breast cancer cohort and specimens used for analysis

Tumor samples were from surgically excised breast tumor specimens obtained from patients enrolled prospectively at two tertiary‐care hospitals (St. John's Medical College and Hospital and Rangadore Memorial Hospital) in Bangalore, from June 2008 to February 2013. Informed consent for use of the material for research was obtained from all patients and the study was approved by the IERB (Institutional Ethics Review Board) at both hospitals. Samples were fixed in 10% neutral buffered formalin at room temperature (RT) and stored as formalin‐fixed paraffin‐embedded (FFPE) blocks. All tissues were sectioned and stained with Hematoxylin and Eosin. Only those with >50% tumor content as estimated by a pathologist (JSP) were chosen for analysis. One hundred and thirty‐four blocks met Quality control (QC) criteria for molecular analysis from 130 patients (4 bilateral tumors). All cases were used for gene expression to assay for miR‐18a expression. Of the 130, 6 cases were lost to follow‐up or could not be staged unambiguously resulting in 124 patients who had complete information and were considered for the computation of survival analysis. The median follow‐up of all 124 patients is 72 months, as of 31 March 2017. All the patients were treated with standard endocrine therapy (tamoxifen or aromatase inhibitors) and stage appropriate chemotherapy. The clinical pathological characteristics of the tumors used for analysis are provided in Table 1.

**TABLE 1:** Clinico‐pathological characteristics of 134 ER‐positive patients: The clinical pathological characteristics of the tumors used for analysis from our case series

	All N (%) (N = 134 patients)
Age (y)	
Mean	59
Median	60
Tumor size (cm)	
Mean	3.1
Median	3
Stage	
I	21 (16)
II	74 (55)
III	39 (29)
Grade	
I	14 (10)
II	68 (51)
III	44 (33)
Nx	8 (6)
Lymph node status	
Positive	44 (33)
Negative	84 (63)
Nx	6 (4)
Menopausal status	
Pre	30 (22)
Post	104 (78)

### 2.2 Quantitative real‐time PCR

Quantitation of RNA, cDNA synthesis, and qRT‐PCR experiments were performed as reported previously.^18^, ^19^ Primers for all genes were designed using the software Primer3Plus and manufactured by Eurofins, Bangalore, India. The primer sequences for the genes tested are given in Table S1.

### 2.3 miRNA quantitation and analysis

miRNA present in total RNA was extracted and converted to cDNA using Stem‐loop primers specific for the chosen miRNA according to published protocols. The Taqman MicroRNA Reverse Transcription Kit (Applied Biosystems, #4366596) was used for cDNA conversion. Concentration of 5‐50 ng/µL of total RNA was used for the conversion of miRNA to cDNA according to manufacturer's instructions using Veriti 96‐well thermal cycler (Applied Biosystems). Taqman MicroRNA inventoried assays for qRT‐PCR (Applied Biosystems, #4427975) were used for each of the test and control miRNA. The assay IDs for each of the tested miRNAs are given in Table S2. The Ct values obtained were normalized with RNU48 and it was selected as an endogenous control owing to its high abundance and least variability when tested across human tissues and cell lines.

### 2.4 Cell lines, culture, and transfection with miR‐18a mimics

The breast cancer cell line MCF7 was obtained from American Type Culture Collection Manassas, VA. The culture conditions and the phenotypic characterization of the cell line have been reported previously.^20^ ZR‐75‐1 was obtained from NCCS (Pune, India where cell authentication was done using STR profiling) and cultured in RPMI supplemented with 10% FBS. Cell lines were recharacterized phenotypically by immunofluorescence in the laboratory and routinely tested for mycoplasma by using the fluorescent dye Hoechst 33 342 on every revival and were found to be negative. For all experimental work using cell lines, a passage number below 20 was used. micrON™ mimic for miR‐18a was purchased from Guangzhou RiboBio Co., Ltd. The mimic was transfected into cultured MCF7 and ZR‐75‐1 using Lipofectamine RNAiMAX Transfection Reagent according to the manufacturer's protocol. Briefly, 0.4 × 10^6^ cells were seeded in a 6‐well plate in antibiotic free media with 10% FBS. The following day micrON^TM^ hsa‐miR‐18a‐5p mimic (hsa‐miR‐18a‐5p MIMAT0000072UAAGGUGCAUCUAGUGCAGAUAG) or micrON^TM^ mimic negative control (>cel‐miR‐239b‐5p MIMAT0000295UUUGUACUACACAAAAGUACUG) were mixed with riboFECT™ CP Buffer. A nonspecific miRNA mimic was used as the scrambled or negative control. The final concentration of the mimic and scrambled was 50 nmol/L. To this complex 12 µL Lipofectamine RNAiMAX was added and incubated for 45 minutes. The transfection complex was then added to the cells along with antibiotic free media with 10% FBS and full distribution over the plate surface was ensured. The cells were incubated for a period of 72 hours before harvesting. The cells overexpressing miR‐18a will be referred to hereafter as MCF7‐miR‐18a‐mimic or ZR‐miR‐18a‐mimic and the cells transfected with the negative control will be referred to as MCF7‐negative control or ZR‐negative control. The transfection efficiency was evaluated by assessing the levels of miR‐18a by q‐PCR and levels of the microRNA targets by blot after 72 hours.

### 2.5 Western blot

The transfected cells were lysed, and the protein expression was assayed as reported previously.^19^ The list of antibodies used, and dilutions are listed in (Table S3). A part of the membrane was reprobed with mouse anti‐β‐actin antibody as a loading control. Immunoreactive bands were visualized using enhanced chemiluminescence. Densitometric analysis was performed using quantity one software (Bio‐rad) as reported previously.^19^

### 2.6 Evaluation of proliferative ability

2 × 10^4^ cells were seeded in 96‐well plates and transfected as described above. Cell proliferation was assessed by MTT assay as described previously.^19^ The assay was performed immediately after transfection (0 hour), 24, 48, 72, 96, and 120 hours post‐transfection. In an individual experiment proliferation was studied in sextuplicates and the overall experiment repeated thrice.

### 2.7 Microarray analysis

Microarray‐based global gene expression analysis of hsa‐miR‐18a‐5p overexpressing MCF7 cells was performed using 8 × 60 v3 Agilent arrays (Genotypic, Technology). Agilent's Quick‐Amp labeling kit was used and one‐color microarray‐based gene expression analysis was performed. The microarray images were manually verified and found to be devoid of uneven hybridization, streaks, blobs, and other artifacts. Microarray data analysis done conforms to the Minimum Information About a Microarray Gene Experiment guidelines. Microarray analysis was carried out using limma package (version: 3.30.13) available from the Bioconductor project. Background correction followed by quantile normalization was performed among microRNA overexpressed and control samples. LmFit and contrasts.fit functions were used to fit a linear model to the normalized expression values of each gene. To obtain more precise estimates of gene‐wise variability, empirical Bayes moderation was carried out by borrowing information across all genes. From the model, differentially expressed genes were filtered based on Benjamini‐Hochberg FDR < 0.05. To the FDR‐filtered data, log2 fold change of greater than 1 and log2 fold change of less than −1 were applied for obtaining overexpressed and underexpressed genes respectively for microRNA overexpressed group. Heatmap of differentially expressed genes was obtained using g plots (version 3.0) R package. Functional enrichment and pathway analysis of overexpressed and underexpressed genes were performed using ToppGene suite.

### 2.8 Wound healing assay

MCF7 cells were transfected as described above. After 72 hours the media were replaced with low serum media (0.2% Fetal Bovine Serum) for 24 hours. The following day, the cell layer was scratched using a pipette tip where adherent cells were observed, and images were captured at the initiation time 0 hour and after 24 hours. The migratory ability was quantified and normalized by relative gap distance and compared between cells transfected with micrON^TM^hsa‐miR‐18a‐5p mimic and micrON^TM^ mimic negative control.

### 2.9 Immunofluorescence

Cells were grown in 8‐well slide chambers and transfected as described above. Immunofluorescence was performed as reported previously.^19^ The cells were incubated in primary antibody anti‐E‐cadherin (Abcam; Rabbit monoclonal EP700Y −1:500) overnight at 4°C and then labeled with the secondary antibody Alexa Fluor 488 Donkey Anti‐Rabbit IgG (H + L) (Invitrogen). The slide was then mounted on gold antifade reagent with DAPI and examined under a fluorescent microscope (Olympus BX51).

### 2.10 Quantitation of F‐actin and G‐actin fractions

G‐actin and F‐actin proteins were separated according to a previously reported protocol.^21^, ^22^ Briefly, transfected cells were washed with PBS, scraped and pelleted. The pellets were resuspended in 50 µL of Triton extraction buffer (0.1% Triton in PBS with proteinase inhibitor cocktail) and incubated on ice for 5 minutes with slight agitation. The samples were then centrifuged at 12,000 rpm at 4°C for 6 minutes. The supernatant (soluble fraction with globular actin) obtained after centrifugation was separated and the pellet with triton insoluble F‐actin component was subjected to lysis with RIPA buffer on ice for 30 minutes with slight agitation and extracted as described before.^19^ An equal amount of soluble and insoluble fractions was loaded on a 12% polyacrylamide gel and western blot was performed as described previously. Percentage change of actin components was calculated using GAPDH used as loading control for the soluble fraction (G‐actin). G‐actin band density was normalized with GAPDH and ratio calculated with respect to F‐actin band density.

### 2.11 Statistical analysis

Descriptive statistics was used for all clinical variables. Difference in the levels of gene and protein expression was evaluated by Mann‐Whitney *U* test or two‐tailed student's *t* test. Correlations were evaluated by Pearsons's correlation coefficient. Kaplan‐Meier analysis was used to examine the estimated differences in disease‐free survival between the miR‐18a high and miR‐18a low groups. Disease‐free survival was calculated as the time from the date of first diagnosis to the time when either a local recurrence or a distance metastasis occurred. Patients without an event or had succumbed to nonbreast cancer‐related causes were right censored. Log‐rank test (Mantel‐Cox) was used to compare the survival between groups. Both univariate and multivariate Cox‐proportional hazard analysis were done to validate the prognostic importance of miR‐18a in comparison to other clinico‐pathological characteristics. For in‐vitro graphical representations, the results are depicted as mean ± standard error of mean or standard deviation calculated from three experiments. Statistical analysis was performed by the Student's *t* test. For all in‐vitro experiments, a value of *P* < .05 was considered to be statistically significant. All statistical analysis was carried out using the software XLSTAT 2015 (Windows).

## 3 RESULTS

### 3.1 miR‐18a expression is higher in tumors with lower ER protein expression and levels are negatively associated with *ESR1* and *GATA3* transcripts

**FIGURE 1:**
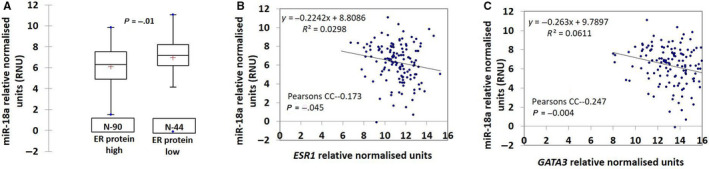
Negative correlation of miR‐18a with ER protein, ESR1, and GATA3 transcripts (A). Correlation of miR‐18a levels with ER protein in ER+ tumors (Samples were segregated into high and low ER‐positive groups using Allred score 7 and above as cut off for the high ER group). (B and C) Correlation of transcript levels of miR‐18a with *ESR1* and *GATA3* transcript levels

hsa‐miR‐18a‐5p is known to be a repressor of ERα expression and enhanced expression has been reported in triple negative tumors.^11^, ^23^ We wanted to estimate the relative abundance of miR‐18a in 134 ER‐positive primary breast cancers from our case series of tumors and found that within ER‐positive samples the transcript levels of the microRNA was highest in the group of tumors that had the least expression of ER protein (*P* = .01; Figure 1A). Samples were segregated into high and low ER‐positive groups using Allred score 7 and above as cut off for the high ER group. Moreover, the levels of hsa‐miR‐18a‐5p also had a negative correlation with transcript levels of ESR1 (*P* = .045; Figure 1B). The same was also seen within ER‐positive samples of the TCGA and the METABRIC cohorts (Figure S1A,D, respectively). GATA3 is a transcriptional factor that is highly expressed in the luminal subtype of breast cancer^24^ and we observed that miR‐18a levels correlated negatively with *GATA3* (*P* = .004) as well (Figure 1C).

### 3.2 Overexpression of miR‐18a in MCF7 decreases ER and its downstream genes; increases proliferative ability and levels of basal cytokeratin 14

To further probe the role of miR‐18a in invasion and metastasis, it was overexpressed using a synthetic mimic for hsa‐miR‐18a‐5p in ER+ cell lines MCF7 and ZR‐75‐1. The expression of miR‐18a‐5p following transfection was found to be increased by a very high fold change (∆∆Ct of 13.7 for MCF7 AND 14.8 for ZR‐75‐1; *P* < .05). There was no change in the levels of other random microRNAs assayed for; hsa‐miR‐21‐5p, hsa‐miR‐182‐5p, and hsa‐miR‐155‐5p between the overexpressed cells and the mimic negative control cells (Figure S2A,E) suggesting successful overexpression of only miR‐18a. Overexpression decreased the transcript levels of miR‐18a targets CDK19 and DICER in MCF7‐miR‐18a‐mimic by twofold (*P* < .05; Figure S2B). We also measured the protein levels of TNFAIP3 which is an experimentally validated target of miR‐18a^25^ to assess the efficacy of overexpression. Overexpression of the microRNA brought down the protein levels by 42% (*P* = .008; Figure S2C,D). ER protein levels decreased by 30% in MCF7 (*P* = .002) (Figure S2C,D) and 43% in ZR‐75‐1 (*P* = .0002; Figure S2G,H) indicating the efficacy of microRNA overexpression. To rule out nonspecific effects of miRNA overexpression, the levels of TGF‐β; a target of miR‐21 was assessed for and no change was recorded in the levels of TGF‐β protein post‐transfection with miR‐18a mimic (Figure S2C,D).

**FIGURE 2:**
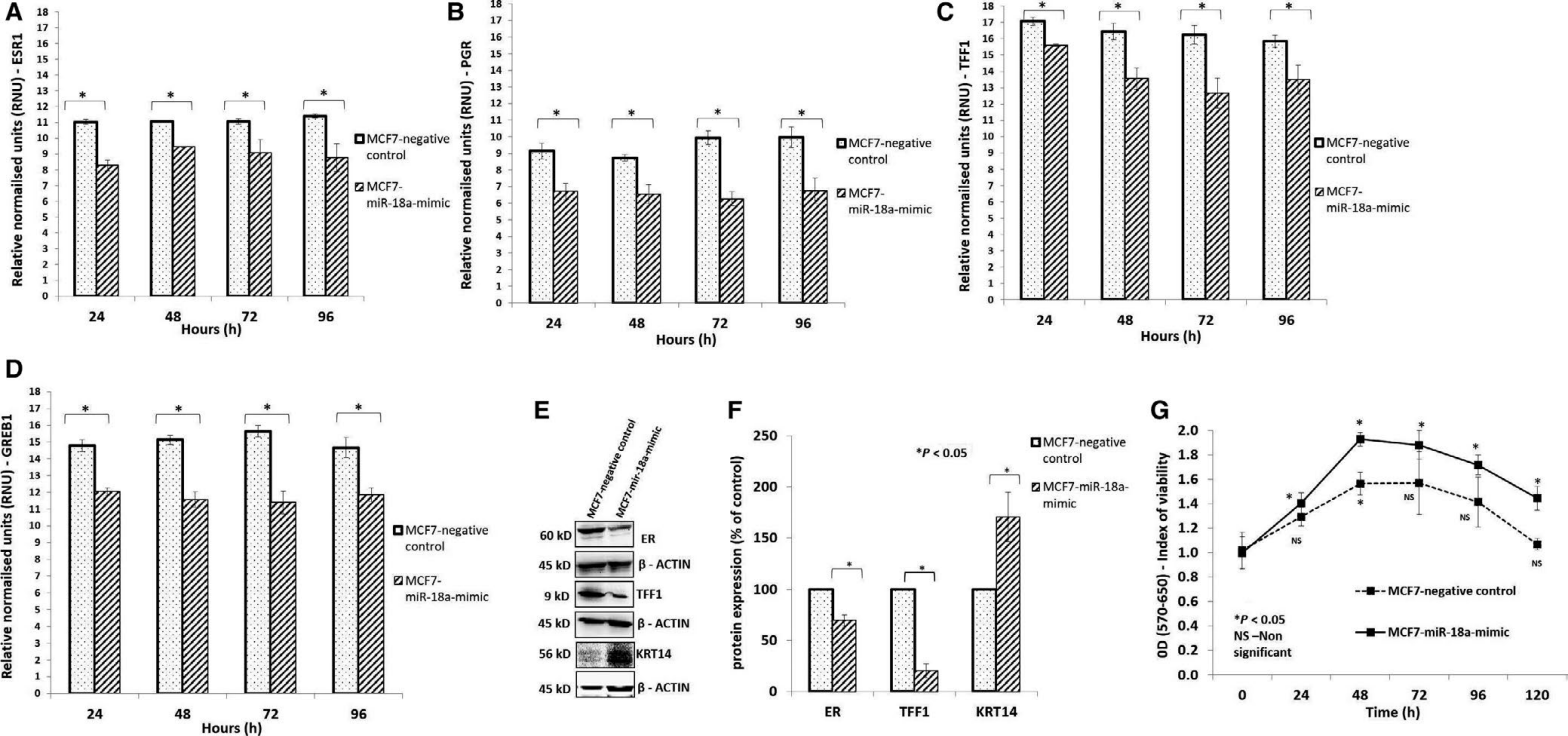
Overexpression of miR‐18a in MCF7 increases basal like phenotype (A‐D). Expression levels of *ESR1, PGR, GREB1,* and *TFF1* transcripts respectively in MCF7‐miR‐18a‐mimic assayed across multiple time points (24‐120 h). E and F, Expression levels of ESR1, TFF1, and KRT14 in MCF7‐miR‐18a‐mimic. G, Proliferation assay done in MCF7‐miR‐18a‐mimic compared to MCF7‐negative control. Assay was done across multiple time points starting from 0 to 120 h. Values are mean ± SEM (n = 3). Statistical analysis was performed by the Student's *t* test compared with the mimic negative control.*P < .05 compared with the vehicle and ^NS^ *P* > .05 (Not significant)

Post‐transfection the levels of ESR1 and downstream genes were assessed for at multiple time points from 24 to 96 hours. We observed a consistent decrease in the expression of genes associated with a “luminal” phenotype (*ESR1*, *PGR*, *TFF1*, *GREB1*; *P* < .05) across all the time points in MCF7‐miR‐18a‐mimic (Figure 2A‐D). The transcript levels of GATA3 and FOXA1 did not change post‐transfection (Data not shown). TFF1 is a small cysteine‐rich secreted protein that is frequently expressed in breast tumors under the control of estrogen. It is a downstream target of ER signaling and hence levels were assessed to measure extent of ER suppression and decrease in luminal features. There was a significant loss of *TFF1* expression of up to 80% in MCF7 (*P* = .0001; Figure 2E,F) and up to 53% in ZR‐75‐1 (*P* < .0001; Figure S2G,H). The transcript levels of basal marker cytokeratin 14 increased in both the cell lines (Figure S3). Protein levels increased by 70% in MCF7 (*P* < .02; Figure 2E,F) accompanied by an increase in proliferative rate of up to 35% (*P* < .05) assessed every 24 hours post‐transfection up to 5 days (Figure 2G). The results indicate that the presence of high levels of miR‐18a in ER‐positive breast cancer cells induces a shift in phenotype of being more proliferative and less of luminal features.

### 3.3 Overexpression of miR‐18a in MCF7 decreases E‐cadherin levels, increases migratory ability, and expression of ECM‐associated genes

**FIGURE 3:**
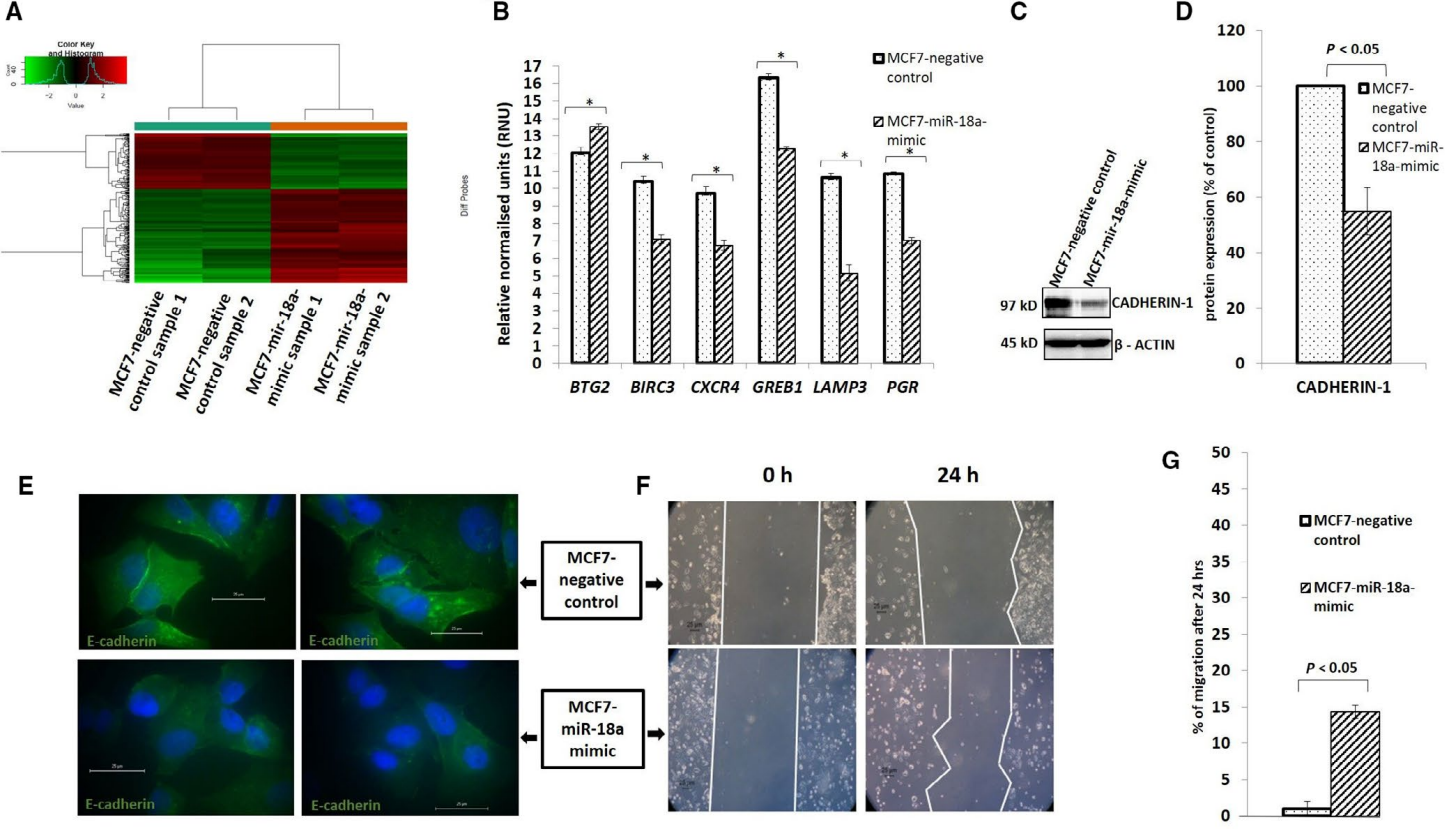
Overexpression of miR‐18a increases migratory ability (A). Heat map for the microarray analysis done on in MCF7‐miR‐18a‐mimic vs MCF7‐negative control. b.Validation by qRT‐PCR of expression levels of genes that were upregulated (*BTG2*) and downregulated (*BIRC3*, *CXCR4*, *GREB1*, *LAMP3,* and *PGR*) in MCF7‐miR‐18a‐mimic as reported in the microarray analysis. C‐E, Levels of E‐cadherin in MCF7 after induction of miR‐18a as reported by blot and immunofluorescence, respectively. F and G, Wound healing assay done on in MCF7‐miR‐18a‐mimic and average percentage of migration calculated from three independent experiments. Values are mean ± SEM (n = 3). Statistical analysis was performed by the Student's *t* test compared with the mimic negative control.*P < .05 compared with the vehicle and ^NS^ *P* > .05 (Not significant)

To identify putative target genes of miR‐18a and the specific pathways upregulated by overexpression of the microRNA, we carried out mRNA microarray analysis of RNA isolated from MCF7‐miR‐18a‐mimic (Figure 3A). The results from microarray were validated by qRT‐PCR analysis for representative mRNAs that were upregulated (BTG2; *P* < .05) or downregulated (BIRC3, CXCR4, GREB1, LAMP3, and PGR; *P* < .05) in the analysis (Figure 3B). The data suggested an increase in the expression of ECM‐associated genes. Analysis of microarray data demonstrated enrichment in pathways that are associated with the Cadherin and the Wnt signaling pathways (Table 2). High levels of miR‐18a also brought about a repression in the levels of Cadherin‐1 (E‐cadherin) up to 45% (*P* = .0004) as demonstrated by blot and immunofluorescence (Figure 3C‐E). E‐cadherin repression in ZR‐75‐1 was 50% (*P* = .0002) as shown in Figure S4A,B. We also observed an increase in the migratory ability up to 15% (*P* < .05) in MCF7‐miR‐18a‐mimic as seen by the wound healing assay (Figure 3F,G). An interesting observation was the increased rate of single cell migration in MCF7‐miR‐18a‐mimic as compared to a visible collective cell migration in MCF7‐negative control.

**TABLE 2:** Enriched events in MCF7‐miR‐18a‐mimic: Enriched events and upregulated pathways from the microarray analysis performed on MCF7‐miR‐18a‐mimic

Pathway	*P* value	Genes from input
Ensemble of genes encoding extracellular matrix (ECM) and ECM‐related proteins	1.535E‐6	18
Genes related to Wnt‐mediated signal transduction	7.826E‐5	5
Cadherin signaling pathway	1.345E‐4	6
Ensemble of genes	2.882E‐4	12

### 3.4 High levels of miR‐18a activate the Wnt pathway and induce actin remodeling via modulating the planar cell polarity pathway in ER‐positive breast cancer cell lines

**FIGURE 4:**
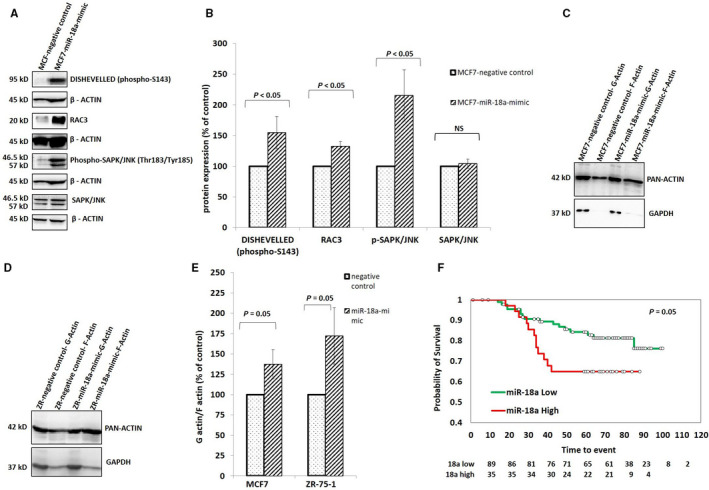
Overexpression of miR‐18a induces cell polarity changes by activating the Wnt pathway (A and B). Expression levels of various proteins that get activated in the PCP pathway—DISHEVELLED (phospho‐S143), RAC3, Phospho‐SAPK/JNK (Thr183/Tyr185), SAPK/JNK in MCF7‐miR‐18a‐mimic c‐e. Polarity changes as recorded by measuring the levels of pan actin in differentially separated G‐ and F‐actin fractions in MCF7 (Fig c) and ZR‐75‐1 (Fig d) post‐transfection, percentage change of G‐actin and F‐actin components recorded as average of three independent experiments (Fig e). Values are mean ± SEM (n = 3). Statistical analysis was performed by the Student's t‐test compared with the mimic negative control. *P < .05, NS *P* > .05 (Not significant). f. Separation of disease‐free survival within the ER+ HER2‐ tumors based on miR‐18a levels in our case series of tumors

As the microarray results showed an upregulation of the Wnt pathway genes such as Wnt11; a noncanonical Wnt pathway gene, we wanted to probe the involvement of the noncanonical Wnt pathway in the cells with overexpressed miR‐18a. We first confirmed the activation of Wnt pathway by assessing the levels of p‐dishevelled; a key component of Wnt signaling pathway that relays Wnt signals from receptors to effectors to facilitate the noncanonical Wnt‐Frizzled/Planar Cell Polarity pathway signaling.^26^ We observed that the levels of p‐Dvl increased in MCF7‐miR‐18a‐mimic by 54% (*P* = .05; Figure 4A,B) and ZR‐miR‐18a‐mimic by 42% (*P* = .08; FIGURE S4C,D). This result confirms the activation of the noncanonical Wnt pathway in these cells post‐transfection. Since the other key mediators of the PCP pathway that mediate regulation of cytoskeleton to change cell polarity^27^ include RAC and JNK, the levels of these proteins were measured. The levels of RAC3 increased by 32% in MCF7‐miR‐18a‐mimic (*P* = .008), p‐SAPK/JNK increased by 115% (*P* = .02) with no changes in the levels of total SAPK/JNK in MCF7 (Figure 4A,B). Since activation of JNK through the PCP pathway induces changes to cell polarity and actin remodeling^28^ we wanted to measure the change in actin dynamics. Hence, we estimated the change in the ratio of G‐actin to F‐actin in MCF7‐miR‐18a‐mimic and ZR‐miR‐18a‐mimic. Triton‐X fractionation and separation of the soluble and insoluble protein fractions revealed a 37% increase (*P* = .05) in the F‐actin fraction in MCF7‐miR‐18a‐mimic and 72% increase in ZR‐miR‐18a‐mimic (*P* = .05; Figure 4C‐E). The results indicate existence of a significant shift in actin dynamics brought about by the high levels of miR‐18a that may aid in cell migration.

To examine if the results seen above were produced due to suppression of ER alone, we accessed the microarray data from previously published experimental study [GSE37820] where estrogen receptor alpha (ESR1) was knocked down in MCF7 breast cancer cells using siRNA. Expression levels of genes that are involved in Wnt noncanonical signaling; WNT11, FZD8, WNT2B, TLE2, and DIXDC1 were compared between MCF7 with ESR1 knock down and nontargeting control treated cells. The analysis revealed that there was no significant change between the levels of the genes after ER silencing (FIGURE S5) indicating these effects were not due to ER silencing alone.

Furthermore, to examine if the results were influenced by combined effect of miR‐18a overexpression with ER suppression, we analyzed the breast cancer data set from TCGA (https://tcga‐data.nci.nih.gov/tcga/). Only hormone receptor positive (ER and PR+) tumors were divided into two groups as miR‐18a high and low based on the cut off value of miR‐18a transcript levels at the third quartile. This segregation identified 192 tumor samples with high miR‐18a similar to the experimental condition of miR‐18a overexpression. These samples were further segregated into ESR1 transcript high (n = 143) and low (n = 49) groups by taking a cut off for ESR1 transcript levels at the third quartile. The expression levels of the genes that are involved in Wnt noncanonical signaling; WNT11, FZD8, WNT2B, TLE2, and DIXDC1 were compared between these groups. WNT11 and WNT2B levels were significantly upregulated (*P* = .001 and *P* = .008, respectively) in the ESR1 low group when compared to the high group. This observation mirrors the results obtained from cell lines where miR‐18a overexpression brought about ER suppression and Wnt activation. These results give an indication that ER suppression and Wnt activation are two independent but synergistic events that occur as a result of abundant levels of miR‐18a. ER suppression alone may not cause the activation of Wnt noncanonical signaling but it is suppression mediated by high levels of miR‐18a is critical to activation of Wnt pathway.

### 3.5 High levels of miR‐18a is prognostic within ER‐positive samples

In order to study the prognostic importance of miR‐18a, we performed Kaplan‐Meier survival analysis on our set of tumor samples. In order to maximize the specificity, we chose to take a cut off for miR‐18a transcript at the third quartile (third quartile at 7.7) and divided the samples into miR‐18a high and low. Stratification of the ER+ tumor samples by hsa‐miR‐18a‐5p levels produced significant separation of the groups based on disease‐free survival in our case series [(Hazard Ratio (HR) of 2.097 (0.988‐4.450); log rank *P* = .05)] (Figure 4F). The disease‐free survival at 6‐year median follow‐up dropped from 85% in miR‐18a low tumors to 66% in miR‐18a high tumors. The prognostic value was also validated in all 124 tumors using both univariate and multivariate Cox‐proportional hazard analysis (Table 3). The analysis was also carried out using the METABRIC cohort and the miR‐18a expression could significantly predict disease‐free survival across all ER+ tumors (n = 375; log rank *P* = .02) [HR of 1.4 (1.046‐1.977)] (FIGURE S6 and Tables S4 and S5). These data confirm the prognostic role of miR‐18a in predicting recurrence in ER+ tumors that express higher levels of this miRNA.

**TABLE 3:** Univariate and Multivariate Cox‐proportional hazard analysis: Univariate and Multivariate Cox‐proportional hazard analysis in tumor specimens that belong to our case series that was used for survival analysis

	All; N = 124			
	Univariate		Multivariate	
	HR (95% CI)	*P* value	HR (95% CI)	*P* value
**Age**				
>50	Reference			
<50	1.102 (0.430‐2.383)	.97		
T‐size				
<3 cm	Reference			
>3 cm	1.544 (0.730‐3.266)	.25		
Lymph node status				
N0	Reference			
N1	2.303 (0.799‐6.640)	.12		
N2	1.983 (0.574‐6.851)	.27		
**N3**	**6.292 (1.914‐20.679)**	**.002**	**5.3 (1.380‐20.368)**	**.015**
Stage				
I	Reference			
II	0.915 (0.291‐2.873)	.87		
III	2.036 (0.663‐6.248)	.21		
Grade				
I	Reference			
II	1.3 (0.0‐13.00)	.98		
III	1.5 (0.0‐15.00)	.98		
Menopausal status				
Post	Reference			
Pre	0.861 (0.348‐2.127)	.74		
**miR‐18A RNU**	**2.097 (0.988‐4.450)**	**.05**	**2.09 (0.850‐5.151)**	**.10**

Abbreviation: T‐size, Tumor size.

Values in bold refer to the parameters that emerged statistically significant in the analysis.

## 4 DISCUSSION

Although hormone receptor‐positive breast cancers tend to have the best clinical prognosis among breast cancers, the luminal tumors as a subgroup are known to be the most heterogeneous at the molecular level encompassing both mutational spectrum and gene expression^29^. There is growing interest in the examination of epigenetic alterations on the presumption that these alterations might be more amenable to clinical manipulation. There are multiple reports supporting the designation of an oncomir to miR‐18a belonging to the miR‐17‐92 cluster in both multiple clinical cancers as well as experimental systems.^12^ In particular, experimentally miR‐18a is known to prevent translation of ERα, providing a direct mechanistic link between miR‐18a and ER.^11^ miR‐18a is also reported to suppress Dicer expression in breast cancer cells.^16^ The most interesting aspect of miR‐18a function in our experiments initially emerged from the analysis of microarray data upon miR‐18a overexpression in MCF7 cells. The altered expression of members of the noncanonical Wnt pathway was an unexpected and novel observation. The activation of the Wnt pathway could be mediated by many of the targets of miR‐18a directly or indirectly. Many studies have reported the synergistic crosstalk that exists between Wnt and estrogen receptor signaling.^30^ miR‐18a is known to modulate the expression of STAT3 activity and studies have shown that STAT3 is known to suppress Wnt signaling during adipogenesis.^31^, ^32^ The PCP pathway includes upstream PCP components such as Fat, Atrophin; core PCP components like Frizzled and Dishevelled; and downstream PCP effectors including Rho, Rac, and JNK that change intracellular cytoskeletal rearrangements impacting cellular behavior.^27^, ^28^, ^33^ Since the process of tumorigenesis recapitulates several aspects of developmental biology^34^ it is not surprising that a dysregulated PCP could be paving way for tumor cell migration as well.^35^ The preponderance of experimental work has focused on EMT and its potential contribution to metastasis. The implication of PCP pathway in our data provides an additional angle to understanding cancer cell migration. Given the fact that both EMT and PCP pathways lead to the process of invasion and migration possible interplay or crosstalk between the two should be further explored.

miR‐18a overexpression also led to an increase in proliferative ability accompanied by a decrease in the expression of *ESR1* and downstream targets like *GREB1*, *TFF1* etc The levels of basal cytokeratin 14 increased indicative of a move from a luminal to a more basal aggressive phenotype. While there was indeed a decrease in the time needed for wound closure what was noted in multiple experiments was a tendency for cells to migrate individual as opposed to the general tendency to move en masse. It is noteworthy to mention that the PCP pathway has been implicated in the directional migration of individual cells by regulation of actin dynamics within short‐lived cellular protrusions.^28^

The fact that ER‐positive breast cancer stratified by miR‐18a levels had differential risk of recurrence not only in our small data set but also the much larger data set of the METABRIC study lends support to the idea that it might indeed be clinically relevant. ER‐positive samples of the TCGA and the METABRIC cohorts also showed a positive correlation between miR‐18a and *RAC3* and *MMP9* (Figure S1B,C,E,F). Thus, the results suggest that miR‐18a may play a role in aiding tumor invasion in ER+ tumors by activating a signaling pathway that can bring about alterations in cell polarity. Exciting new developments in the area of drug discovery and clinical development of molecules targeting these pathways make these experimental observations interesting both from a molecular mechanistic view as well from the viewpoint of potential clinical utility.

## CONFLICT OF INTERESTS

The authors have no conflict of interest.

## AUTHORS' CONTRIBUTIONS

MGN was involved in design of the work, the acquisition, analysis, and interpretation of data, and has drafted the work; JSP and STS were involved in interpretation of data and has drafted the work; AK, SR, HPS, RK, AA, and RR were involved in the acquisition and analysis of data; SBS provided clinical samples; All authors have read and approved the final manuscript.

## ETHICAL APPROVAL

All procedures performed in the studies involving human participants were in accordance with the ethical standards of both St. John's Medical College and Hospital (No. 62/2008) and Rangadore Memorial Hospital (RMHEC/02/2010) and with the 1964 Helsinki declaration and its later amendments or comparable ethical standards.

## Supporting information

Figure S1

Figure S2

Figure S3

Figure S4

Figure S5

Figure S6

Supplementary Material

## Data Availability

I confirm that my article contains a Data Availability Statement even if no data are available (list of sample statements) unless my article type does not require one. I confirm that I have included a citation for available data in my references section, unless my article type is exempt.

## References

[1] Bray F , Ferlay J , Soerjomataram I , Siegel RL , Torre LA , Jemal A . Global cancer statistics 2018: GLOBOCAN estimates of incidence and mortality worldwide for 36 cancers in 185 countries. CA Cancer J Clin. 2018;68(6):394‐424. 30207593 10.3322/caac.21492

[2] Murphy CG , Dickler MN . Endocrine resistance in hormone‐responsive breast cancer: mechanisms and therapeutic strategies. Endocr Relat Cancer. 2016;23(8):R337‐R352. 27406875 10.1530/ERC-16-0121

[3] Hart CD , Migliaccio I , Malorni L , Guarducci C , Biganzoli L , Di Leo A . Challenges in the management of advanced, ER‐positive, HER2‐negative breast cancer. Nat Rev Clin Oncol. 2015;12(9):541‐552. 26011489 10.1038/nrclinonc.2015.99

[4] Zhang J , Zhou C , Jiang H , et al. ZEB1 induces ER‐alpha promoter hypermethylation and confers antiestrogen resistance in breast cancer. Cell Death Dis. 2017;8(4):e2732. 28383555 10.1038/cddis.2017.154 PMC5477580

[5] Zou Q , Zhou E , Xu F , Zhang D , Yi W , Yao J . A TP73‐AS1/miR‐200a/ZEB1 regulating loop promotes breast cancer cell invasion and migration. J Cell Biochem. 2018;119(2):2189‐2199. 28857253 10.1002/jcb.26380

[6] Agostini M , Knight RA . miR‐34: from bench to bedside. Oncotarget. 2014;5(4):872‐881. 24657911 10.18632/oncotarget.1825 PMC4011589

[7] Shah MY , Calin GA . MicroRNAs miR‐221 and miR‐ 222: a new level of regulation in aggressive breast cancer. Genome Med. 2011;3(8):56. 21888691 10.1186/gm272 PMC3238182

[8] Kong W , He L , Richards EJ , et al. Upregulation of miRNA‐155 promotes tumour angiogenesis by targeting VHL and is associated with poor prognosis and triple‐negative breast cancer. Oncogene. 2013;33:679. 23353819 10.1038/onc.2012.636 PMC3925335

[9] Zhao Y , Deng C , Wang J , et al. Let‐7 family miRNAs regulate estrogen receptor alpha signaling in estrogen receptor positive breast cancer. Breast Cancer Res Treat. 2011;127(1):69‐80. 20535543 10.1007/s10549-010-0972-2

[10] Kondo N , Toyama T , Sugiura H , Fujii Y , Yamashita H . miR‐206 Expression is down‐regulated in estrogen receptor alpha‐positive human breast cancer. Can Res. 2008;68(13):5004‐5008. 10.1158/0008-5472.CAN-08-0180 18593897

[11] Liu WH , Yeh SH , Lu CC , et al. MicroRNA‐18a prevents estrogen receptor‐alpha expression, promoting proliferation of hepatocellular carcinoma cells. Gastroenterology. 2009;136(2):683‐693. 19027010 10.1053/j.gastro.2008.10.029

[12] Yoshimoto N , Toyama T , Takahashi S , et al. Distinct expressions of microRNAs that directly target estrogen receptor alpha in human breast cancer. Breast Cancer Res Treat. 2011;130(1):331‐339. 21755340 10.1007/s10549-011-1672-2

[13] Hsu T‐I , Hsu C‐H , Lee K‐H , et al. MicroRNA‐18a is elevated in prostate cancer and promotes tumorigenesis through suppressing STK4 in vitro and in vivo. Oncogenesis. 2014;3:e99. 24752237 10.1038/oncsis.2014.12 PMC4007194

[14] Luo Z , Dai Y , Zhang L , et al. miR‐18a promotes malignant progression by impairing microRNA biogenesis in nasopharyngeal carcinoma. Carcinogenesis. 2013;34(2):415‐425. 23097559 10.1093/carcin/bgs329

[15] Morimura R , Komatsu S , Ichikawa D , et al. Novel diagnostic value of circulating miR‐18a in plasma of patients with pancreatic cancer. Br J Cancer. 2011;105(11):1733‐1740. 22045190 10.1038/bjc.2011.453 PMC3242609

[16] Sha LY , Zhang Y , Wang W , et al. MiR‐18a upregulation decreases Dicer expression and confers paclitaxel resistance in triple negative breast cancer. Eur Rev Med Pharmacol Sci. 2016;20(11):2201‐2208. 27338043

[17] Leivonen S‐K , Mäkelä R , Östling P , et al. Protein lysate microarray analysis to identify microRNAs regulating estrogen receptor signaling in breast cancer cell lines. Oncogene. 2009;28(44):3926‐3936. 19684618 10.1038/onc.2009.241

[18] Korlimarla A , Prabhu JS , Anupama CE , Remacle J , Wahi K , Sridhar TS . Separate quality‐control measures are necessary for estimation of RNA and methylated DNA from formalin‐fixed, paraffin‐embedded specimens by quantitative PCR. J Mol Diagn. 2014;16(2):253‐260. 24412525 10.1016/j.jmoldx.2013.11.003

[19] Nair MG , Desai K , Prabhu JS , Hari PS , Remacle J , Sridhar TS . beta3 integrin promotes chemoresistance to epirubicin in MDA‐MB‐231 through repression of the pro‐apoptotic protein, BAD. Exp Cell Res. 2016;346(1):137‐145. 27235542 10.1016/j.yexcr.2016.05.015

[20] Nair MG , Desai K , Prabhu JS , Hari PS , Remacle J , Sridhar TS . Data on alteration of hormone and growth factor receptor profiles over progressive passages of breast cancer cell lines representing different clinical subtypes. Data Brief. 2016;8:944‐947. 27508248 10.1016/j.dib.2016.07.001 PMC4961495

[21] Papakonstanti EA , Stournaras C . Association of PI‐3 kinase with PAK1 leads to actin phosphorylation and cytoskeletal reorganization. Mol Biol Cell. 2002;13(8):2946‐2962. 12181358 10.1091/mbc.02-01-0599 PMC117954

[22] Parreno J , Raju S , Niaki MN , et al. Expression of type I collagen and tenascin C is regulated by actin polymerization through MRTF in dedifferentiated chondrocytes. FEBS Lett. 2014;588(20):3677‐3684. 25150168 10.1016/j.febslet.2014.08.012

[23] Calvano Filho CMC , Calvano‐Mendes DC , Carvalho KC , et al. Triple‐negative and luminal A breast tumors: differential expression of miR‐18a‐5p, miR‐17‐5p, and miR‐20a‐5p. Tumour Biol. 2014;35(8):7733‐7741. 24810926 10.1007/s13277-014-2025-7

[24] Voduc D , Cheang M , Nielsen T . GATA‐3 expression in breast cancer has a strong association with estrogen receptor but lacks independent prognostic value. Cancer Epidemiol Biomarkers Prev. 2008;17(2):365‐373. 18268121 10.1158/1055-9965.EPI-06-1090

[25] Trenkmann M , Brock M , Gay RE , Michel BA , Gay S , Huber LC . Tumor necrosis factor alpha‐induced microRNA‐18a activates rheumatoid arthritis synovial fibroblasts through a feedback loop in NF‐kappaB signaling. Arthritis Rheum. 2013;65(4):916‐927. 23280137 10.1002/art.37834

[26] Gao C , Chen YG . Dishevelled: the hub of Wnt signaling. Cell Signal. 2010;22(5):717‐727. 20006983 10.1016/j.cellsig.2009.11.021

[27] Wang Y . Wnt/Planar cell polarity signaling: a new paradigm for cancer therapy. Mol Cancer Ther. 2009;8(8):2103‐2109. 19671746 10.1158/1535-7163.MCT-09-0282

[28] Davey CF , Moens CB . Planar cell polarity in moving cells: think globally, act locally. Development (Cambridge, England). 2017;144(2):187‐200. 10.1242/dev.122804 PMC5394761 28096212

[29] Prat A , Perou CM . Deconstructing the molecular portraits of breast cancer. Mol Oncol. 2011;5(1):5‐23. 21147047 10.1016/j.molonc.2010.11.003 PMC5528267

[30] Gao Y , Huang E , Zhang H , et al. Crosstalk between Wnt/β‐catenin and estrogen receptor signaling synergistically promotes osteogenic differentiation of mesenchymal progenitor cells. PLoS One. 2013;8(12):e82436. 24340027 10.1371/journal.pone.0082436 PMC3855436

[31] Cantwell MT , Farrar JS , Lownik JC , et al. STAT3 suppresses Wnt/beta‐catenin signaling during the induction phase of primary Myf5+ brown adipogenesis. Cytokine. 2018;111:434‐444. 29934048 10.1016/j.cyto.2018.05.023 PMC6289720

[32] Wu W , Takanashi M , Borjigin N , et al. MicroRNA‐18a modulates STAT3 activity through negative regulation of PIAS3 during gastric adenocarcinogenesis. Br J Cancer. 2013;108(3):653‐661. 23322197 10.1038/bjc.2012.587 PMC3593546

[33] Goodrich LV . The plane facts of PCP in the CNS. Neuron. 2008;60(1):9‐16. 18940584 10.1016/j.neuron.2008.09.003 PMC2833340

[34] Bynum WF . The genesis of cancer. A study in the history of ideas. Med Hist. 1980;24(3):360‐361.

[35] Daulat AM , Borg JP . Wnt/Planar cell polarity signaling: new opportunities for cancer treatment. Trends Cancer. 2017;3(2):113‐125. 28718442 10.1016/j.trecan.2017.01.001

