# Supplementary figures and images for: miR‐18a activates Wnt pathway in ER‐positive breast cancer and is associated with poor prognosis
Source: Cancer Med. 2020 Jun 16;9(15):5587–97. doi: 10.1002/cam4.3183 (PMC7402845; doi:10.1002/cam4.3183)

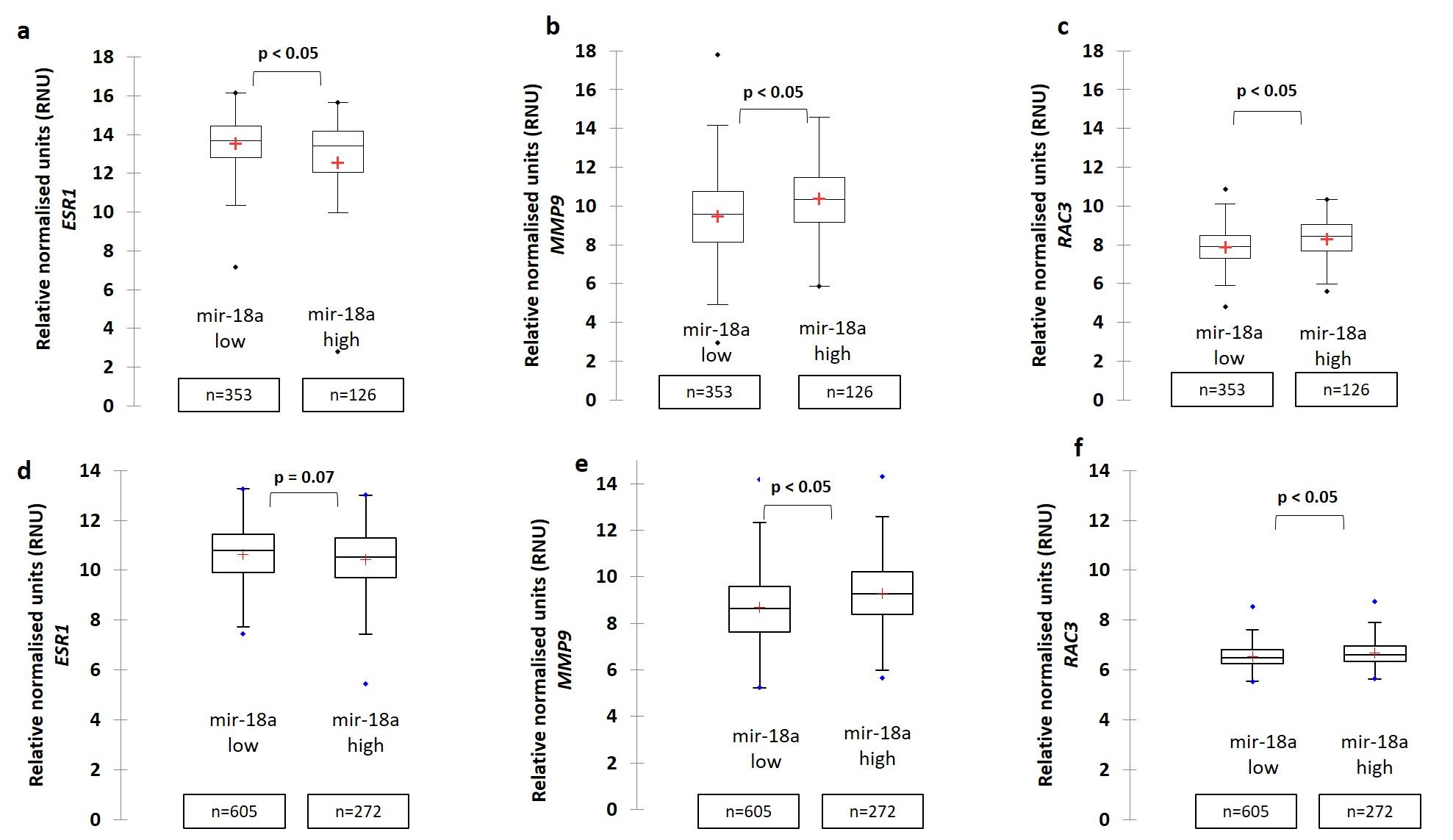

Supplement: Supplementary file 1 — Figure S1 [file CAM4-9-5587-s001.jpg]

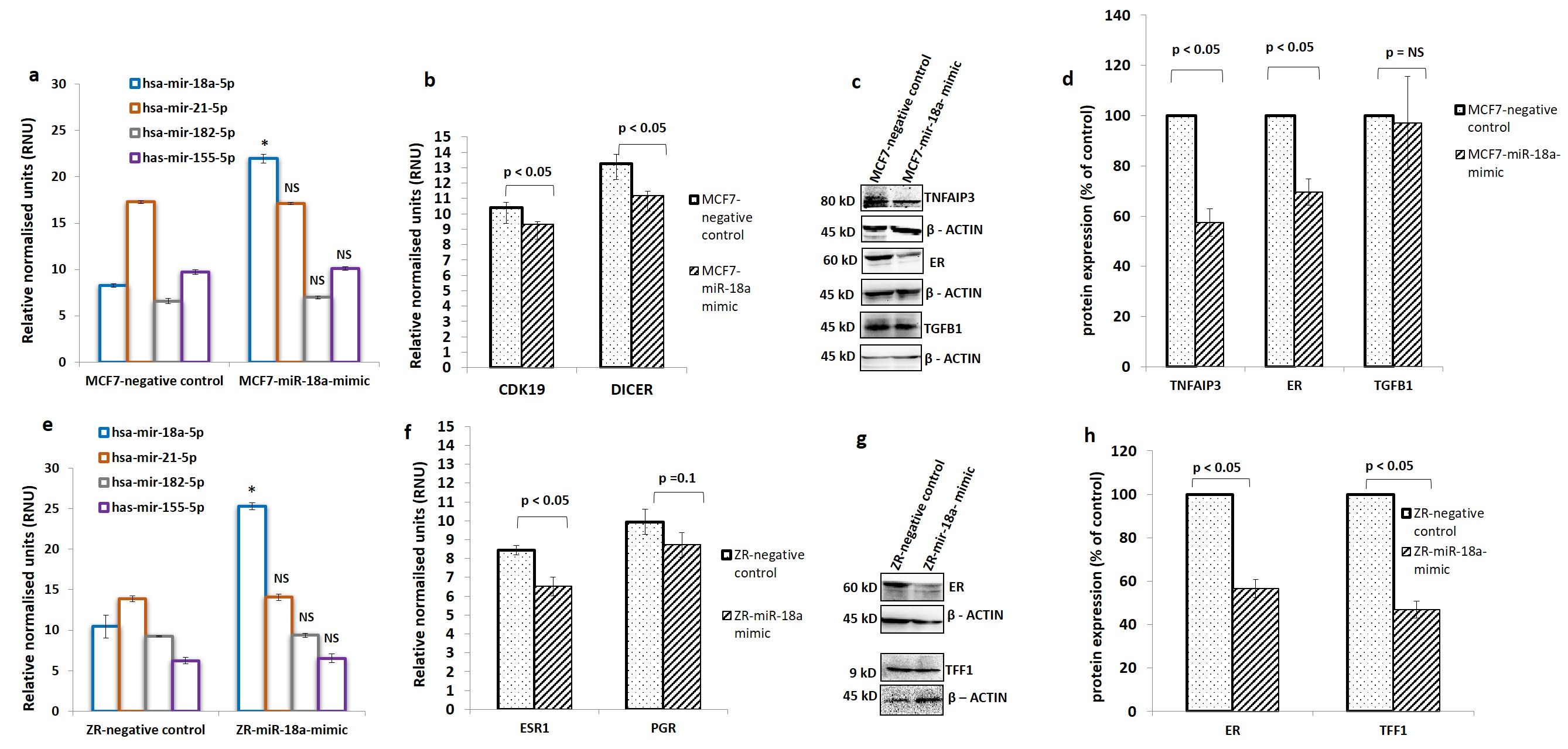

Supplement: Supplementary file 2 — Figure S2 [file CAM4-9-5587-s002.jpg]

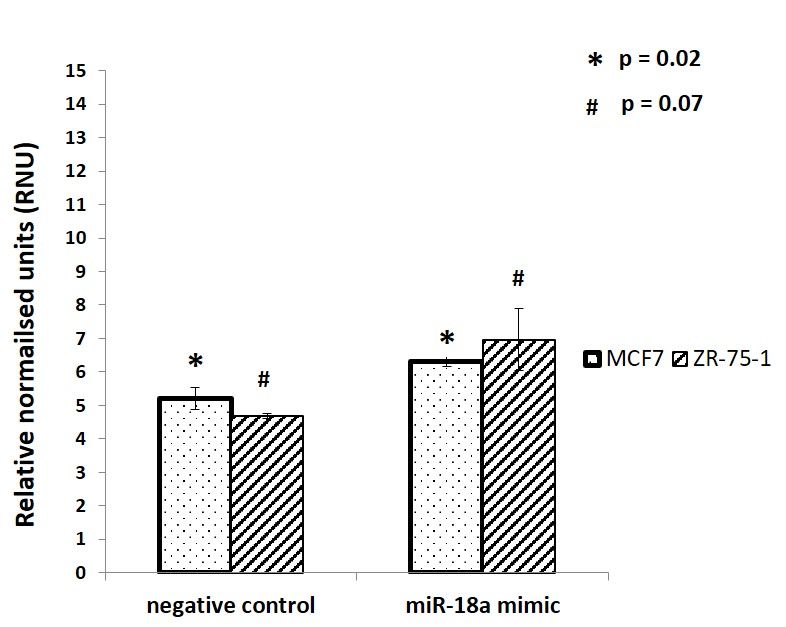

Supplement: Supplementary file 3 — Figure S3 [file CAM4-9-5587-s003.jpg]

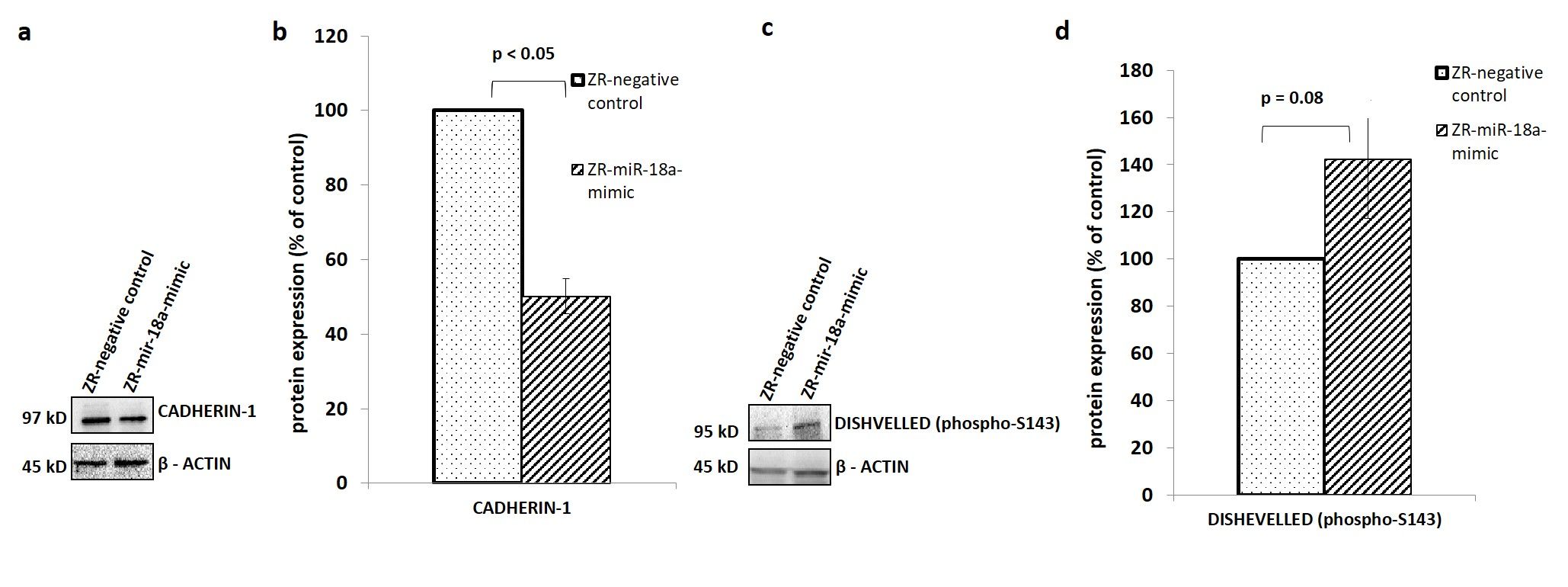

Supplement: Supplementary file 4 — Figure S4 [file CAM4-9-5587-s004.jpg]

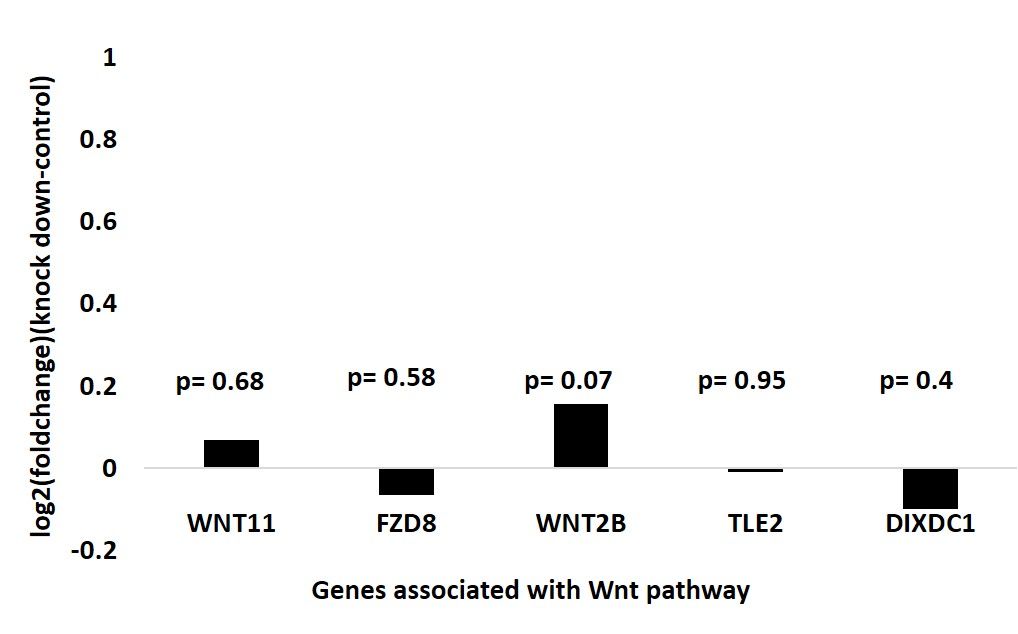

Supplement: Supplementary file 5 — Figure S5 [file CAM4-9-5587-s005.jpg]

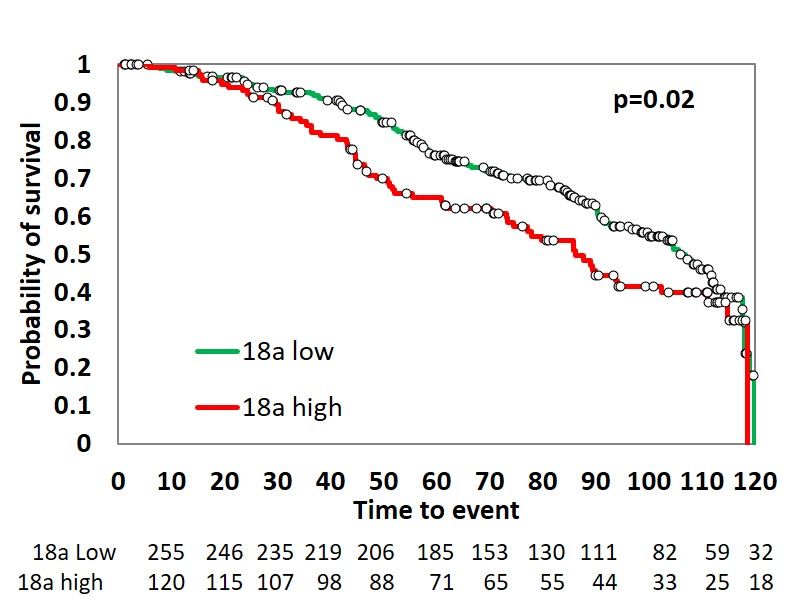

Supplement: Supplementary file 6 — Figure S6 [file CAM4-9-5587-s006.jpg]
