# Supplementary material for: miR‐18a activates Wnt pathway in ER‐positive breast cancer and is associated with poor prognosis
Source: Cancer Med. 2020 Jun 16;9(15):5587–97. doi: 10.1002/cam4.3183 (PMC7402845; doi:10.1002/cam4.3183)
Supplement: Supplementary file 7 — Supplementary Material [file CAM4-9-5587-s007.doc]

**Supplementary data**

**FIGURES:**

**Figure. S1 *TCGA, METABRIC validation for correlation of miR-18a with ESR1, RAC3, MMP9 transcripts*** a & d. Correlation of transcript levels of ESR1 with miR-18a high and low groups in ER+ tumors of TCGA and METABRIC respectively (ER+ tumors were divided into two groups based on the top quartile cut-off value for miR-18a; 3.33 for TCGA and 7.5 for METABRIC datasets). b & e. Correlation of transcript levels of MMP9 with miR-18a high and low groups in ER+ tumors of TCGA and METABIC respectively. c & f. Correlation of transcript levels of RAC3 with miR-18a high and low groups in ER+ tumors of TCGA and METABIC respectively. Statistical analysis was performed by the Mann- Whitney test.


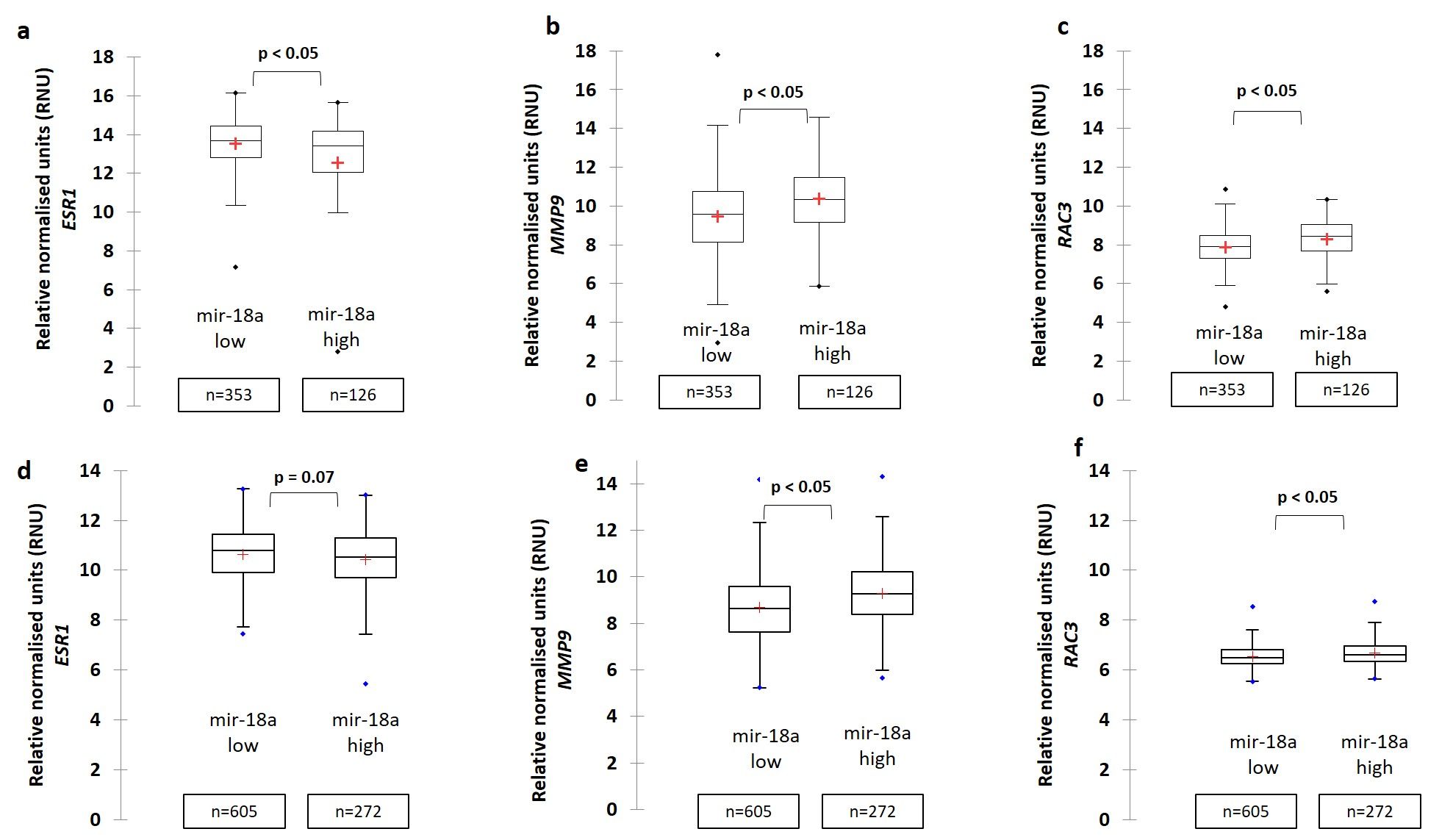


**Figure. S2 *Over-expression of miR-18a in MCF7 and ZR-75-1 decreases level of it’s targets at protein and transcript levels***. a & e. Expression levels of microRNAs – miR 18a, miR-21, miR-182 and miR-155 in MCF7-miR-18a-mimic and ZR-miR-18a-mimic respectively. b. Expression levels of miR-18a targets ; *CDK19* and *DICER* at transcript levels in MCF7-miR-18a-mimic. c & d. Expression levels of TNFAIP3, ESR1 and TGFβ1 proteins in MCF7-miR-18a-mimic. f. Expression levels of *ESR1* and *PGR* at transcript levels post transfection in ZR-75-1. g & h. Expression levels of ESR1 and TFF1 proteins in ZR-miR-18a-mimic. Values are mean ± S.E.M. (n=3). Statistical analysis was performed by the Student’s t-test compared with the mimic negative control. *p<0.05 compared with the vehicle and ^NS^p> 0.05 (Not significant).


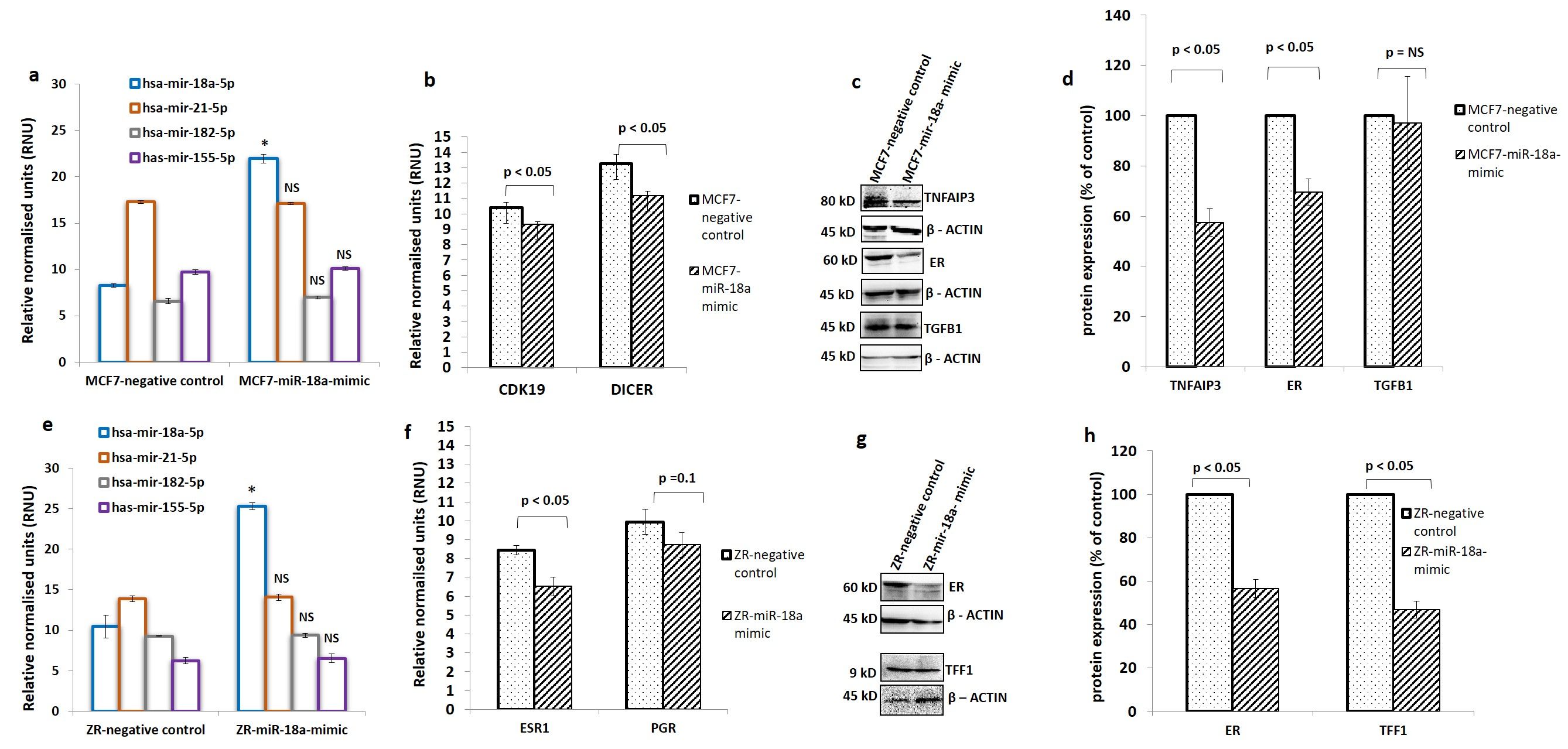


**Figure. S3 *Over-expression of miR-18a in MCF7 and ZR-75-1 increases transcript level of keratin 14.*** Expression levels of keratin 14 transcripts in ZR-miR-18a-mimic and MCF7-miR-18a-mimic vs mimic negative control cells of each cell line respectively. Values are mean ± S.E.M. (n=3) for MCF7 and (n=2) for ZR-75-1. Statistical analysis was performed by the Student’s t-test compared with the mimic negative control. *p<0.05 compared with the vehicle.


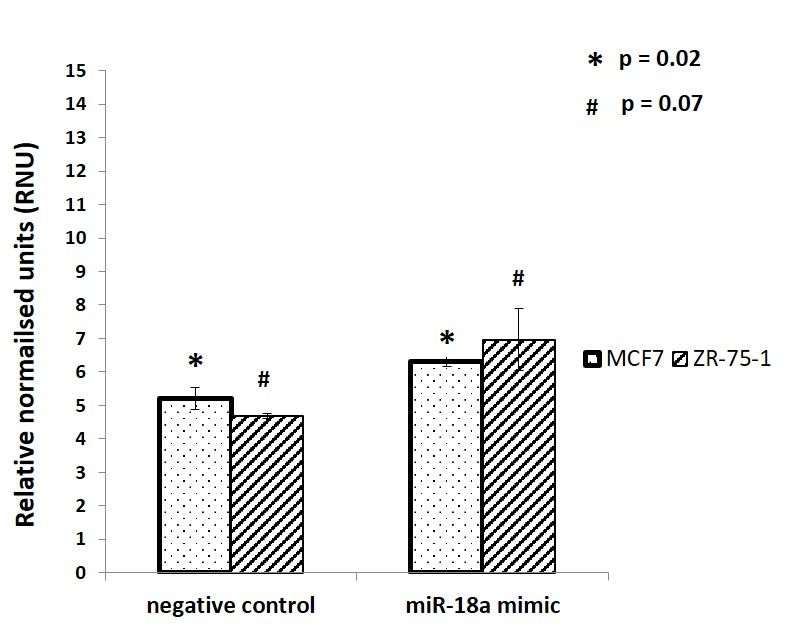


**Figure. S4 *Over-expression of miR-18a in ZR-75-1 decreases level of cadherin-1 and increases p-dishvelled*** a & b. Expression levels of Cadherin-1 in ZR-miR-18a-mimic. c & d. Expression levels of Dishvelled (phosph-S143) in ZR-miR-18a-mimic. Values are mean ± S.E.M. (n=3) for Cadherin-1 and (n=2) for Dishvelled (phosph-S143). Statistical analysis was performed by the Student’s t-test compared with the mimic negative control. *p<0.05 compared with the vehicle.


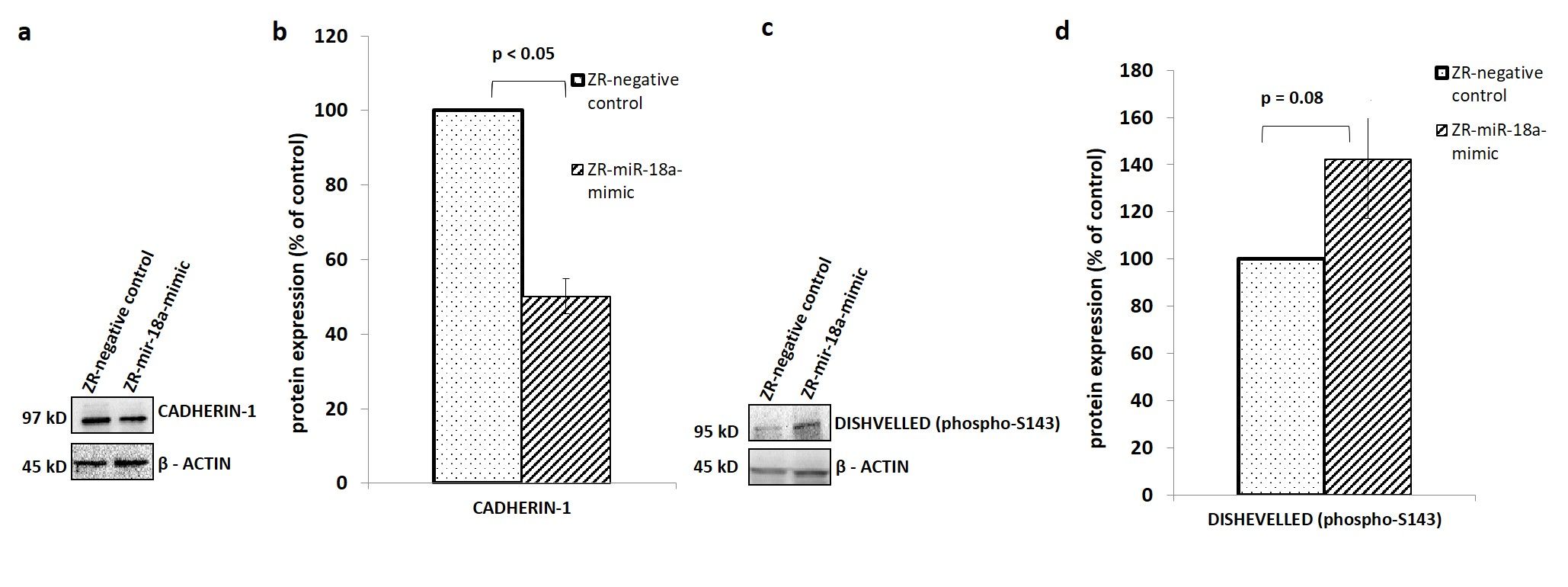


**Figure. S5 *Analysis of the microarray data obtained from GEO-*** ***GSE37820.***

a. Microarray data obtained from GEO (Gene Expression Omnibus) of the study titled “Gene expression profile in MCF7 breast cancer cells after siRNA knock down of estrogen receptor alpha (ESR1)” [GSE37820] where estrogen receptor alpha (ESR1) was knocked down in MCF7 breast cancer cells using siRNA. The gene expression profiles of MCF7 cells, along with non-targeting control treated cells were analysed using Affymetrix Human Genome U133 Plus 2.0 microarrays. The log2 (foldchange) (knock down-control) of various genes associated with the Wnt pathway is plotted with level of significance.


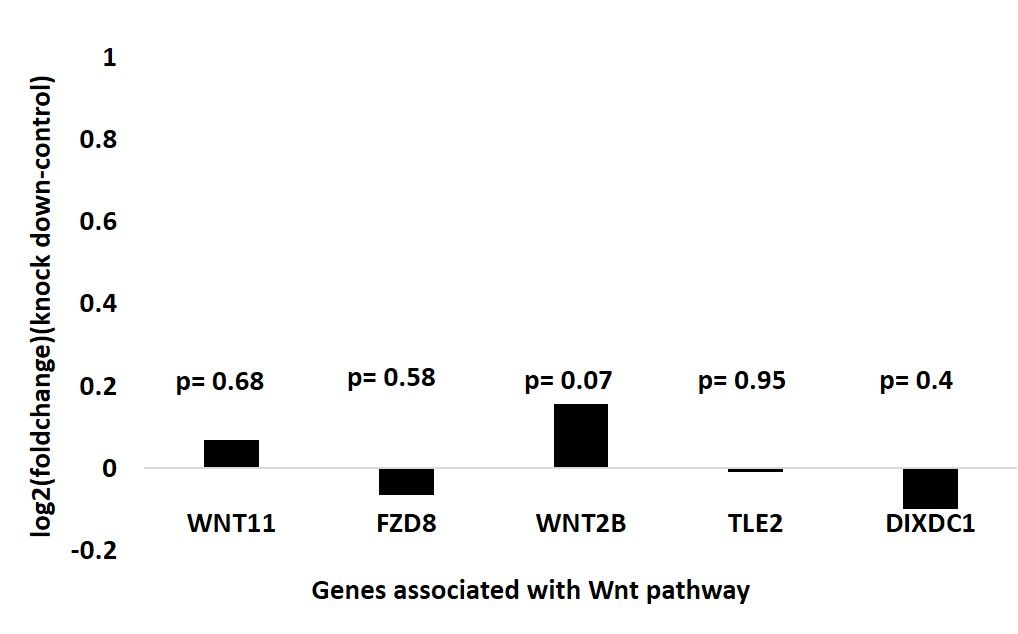


**Figure. S6 *METABRIC validation of miR-18a as a prognostic marker in ER + breast tumors***

a***.*** ER+ tumors were divided into two groups based on the top quartile cut-off value for miR-18a; 7.5. miR-18a levels could separate disease-free survival within the ER+ HER2- tumors by a significant HR of 1.4 (1.046-1.977) log rank p = 0.02. The disease-free survival rate dropped from 60% in miR-18a low tumors to 49.6% in miR-18a high tumors. The prognostic value was also validated in all 375 tumors using both univariate and multivariate Cox-proportional hazard analysis (Supplementary table 5).


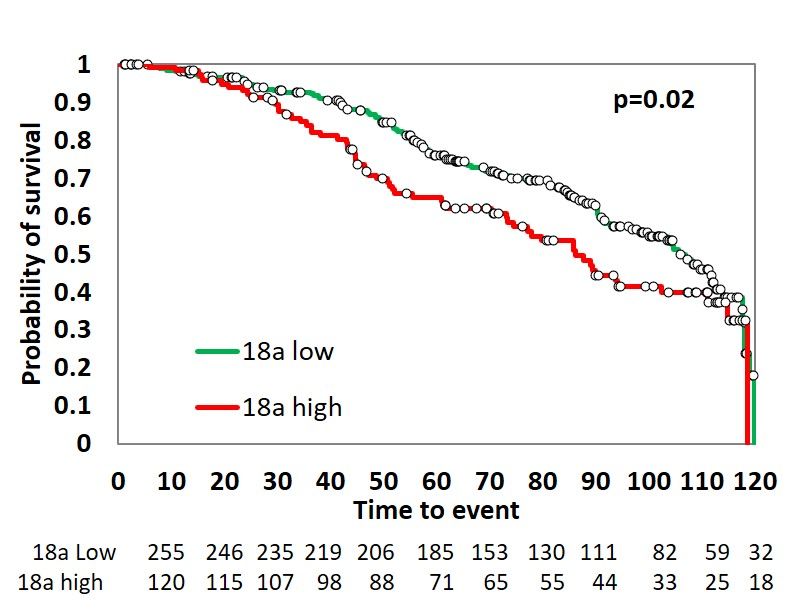


**TABLES:**

**Table S1.** List of primers used for the study

| Gene | Primer Sequence |
| --- | --- |
| *ACTB* | F-TTCCTGGGCATGGAGTC  R-CAGGTCTTTGCGGATGTC |
| *RPLP0* | F-GGCTGTGGTGCTGATGGGCAAGAA  R-TTCCCCCGGATATGAGGCAGCAGT |
| *PUM1* | F-CCGGAGATTGCTGGACATATAA  R-TGGCACGCTCCAGTTTC |
| *ESR1* | F-CCCACCAGAGGCCCTCGAAA  R-AAGCGGGTCACCTGGTCAGT |
| *GATA3* | F-TACCTGAACCGGCACCTG  R-GCCGTACAGTTCCACAAAGG |
| *CDK19* | F-TACCTCCATGCAAATTGGGTGCT  R-TTTGACTCTCCCCCTCTCAGGA |
| *DICER* | F-TTAACCTTTTGGTGTTTGATGAGTGT  R-GCGAGGACATGATGGACAATT |
| *PGR1* | F-TTATAATTCGAGGCGGTTAGTGTTT  R-TCGAACTTCTACTAACTCCGTACTACGA |
| *GREB1* | F-AGACGGGTTTTGCCAGGCCG  R-TGACCCCCACGAGGAGAAAGCC |
| *TFF1* | F-TCTGCCTGCATCCTGACGCGGT  R-AGCGTGTCTGAGGTGTCCGGTC |
| *BTG2* | F-GCACTCACAGAGCACTACAAAC  R-CCATCTTGTGGTTGATGCGAA |
| *BIRC3* | F-CCATGGGTTCAACATGCCAAGTGGT  R-GGGTAACTGGCTTGAACTTGACGG |
| *CXCR4* | F-AACTGGCATTGTGGGCAATGG  R-AGGTGCAGCCTGTACTTGTCCG |
| *LAMP3* | F-GCAGAGATGGGGATACAGCTGATTGT  R-CTTGCGTTGCGTTGGGGTCG |

The details of primers used for gene expression analysis are tabulated along with the primer sequences.

**Table S2.** List of miRNA primer probes used for the study

| Probe | Assay ID |
| --- | --- |
| RNU-48 | RNU48-001006 |
| miR-18a | hsa-miR-18a-002422 |
| miR-21 | hsa-miR-21-00397 |
| miR-182 | hsa-miR-182-002334 |
| miR-155 | hsa-miR-155-002623 |

The details of miRNA primer probes used for gene expression analysis are tabulated along with the assay ID.

**Table S3.** List of antibodies used for western blot

| Antibody | Dilution | Catalogue number |
| --- | --- | --- |
| ER | 1:1000 | ab32063; Abcam |
| TNFAIP3 | 1:1000 | ab92324; Abcam |
| TGF-β 1 | 1:1000 | ab179695; Abcam |
| TFF1 | 1:5000 | ab92377; Abcam |
| CK14 | 1:50 | PM118 ;Pathnsitu |
| E-cadherin | 1:5000 | ab40772; Abcam |
| p-Dishevelled | 1:1000 | ab124933; Abcam |
| Rac3 | 1:5000 | ab124943; Abcam |
| p-JNK/SAPK (Thr183/Tyr185) | 1:1000 | 9251; Cell signalling |
| JNK/SAPK | 1:1000 | 9252; Cell signalling |
| β-Actin | 1:1000 | sc-47778; Santacruz |
| Pan-Actin | 1:1000 | ab14128; Abcam |
| GAPDH | 1:10,000 | EPR16891; Abcam |

The details of antibodies used for western blot analysis are tabulated along with dilutions and product details.

**Table S4.** Clinico-pathological characteristics

|  | **All N (%)**  **(N = 375 patients)** |
| --- | --- |
| **Age (y)** |  |
| Mean | 65 |
| Median | 66 |
|  |  |
| **Tumor Size (cm)** |  |
| Mean | 2.1 |
| Median | 2.5 |
|  |  |
|  |  |
| **Stage**  0 | 90 (24) |
| I | 70 (19) |
| II | 147 (39) |
| III | 28 (7) |
| IV | 7 (2) |
| Nx | 33 (9) |
|  |  |
|  |  |
| **Grade** |  |
| I | 39 (10) |
| II | 170 (45) |
| III | 153 (41) |
| Nx | 13 (3) |
|  |  |
| **Lymph Node status** |  |
| Positive | 171 (46) |
| Negative | 203 (54) |
| Nx | 1 (0) |
|  |  |
| **Menopausal status** |  |
| Pre | 53(14) |
| Post | 322 (86) |
|  |  |

Clinico-pathological characteristics of ER positive patients from METABRIC dataset used for survival analysis.

**Table S5.** Univariate and Multivariate Cox-proportional hazard analysis

|  | **All; N = 375** | | | |
| --- | --- | --- | --- | --- |
|  | **Univariate** | | **Multivariate** | |
|  | HR (95% CI) | p-value | HR (95% CI) | p-value |
|  |  |  |  |  |
| **Age**  >50  <50 | Reference  0.946 (0.591-1.513) | 0.81 |  |  |
|  |  |  |  |  |
| **T-size** |  |  |  |  |
| <=3cm | Reference |  |  |  |
| **>3cm** | **2.471 (1.79-3.410)** | **<0.0001** | **0.436 (0.308-0.618)** | **<0.0001** |
|  |  |  |  |  |
| **Lymph Node status**  N0 | Reference |  |  |  |
| N1 | 1.093 (0.746-1.601) | 0.64 |  |  |
| N2  **N3** | **2.376 (1.588-3.556)**  **2.320 (1.337-4.027)** | **<0.0001**  **0.003** | **1.644 (1.073-2.519)**  **Not significant** | **0.022** |
|  |  |  |  |  |
| **Stage**  0 | Reference |  |  |  |
| I | **0.486 (0.289-0.816)** | **0.006** |  |  |
| II | 0.743 (0.501-1.102) | 0.14 |  |  |
| III  **IV** | 1.297 (0.742-2.268)  **4.698 (2.104-10.489)** | 0.36  **0.000** |  |  |
|  |  |  |  |  |
| **Grade** |  |  |  |  |
| I | Reference |  |  |  |
| II | 1.115 (0.585-2.124) | 0.741 |  |  |
| **III** | **1.898 (1.011-3.562)** | **0.046** | **Not significant** |  |
|  |  |  |  |  |
| **Menopausal status** |  |  |  |  |
| Post | Reference |  |  |  |
| Pre | 0.873(0.557-1.370) | 0.55 |  |  |
|  |  |  |  |  |
| **miR-18A RNU** | **1.438(1.046-1.977)** | **0.02** | **1.328 (0.952-1.851)** | **0.09** |

Univariate and Multivariate Cox-proportional hazard analysis in the METABRIC dataset used for survival analysis.
